# Combined Protocol for Acute Malnutrition Study (ComPAS) in rural South Sudan and urban Kenya: study protocol for a randomized controlled trial

**DOI:** 10.1186/s13063-018-2643-2

**Published:** 2018-04-24

**Authors:** Jeanette Bailey, Natasha Lelijveld, Bethany Marron, Pamela Onyoo, Lara S. Ho, Mark Manary, André Briend, Charles Opondo, Marko Kerac

**Affiliations:** 1 International Rescue Committee, New York, NY USA; 2 Department of Population Health, MARCH Centre, London School of Hygiene and Tropical Medicine, London, UK; 3 No Wasted Lives, Action Against Hunger—UK, London, UK; 4 Action Against Hunger—USA, New York, NY USA; 5 Department of Pediatrics, Washington University School of Medicine in St. Louis, St. Louis, MO USA; 6 Department of International Health, University of Tampere, Tampere, Finland; 7 Department of Nutrition, Exercise and Sports, University of Copenhagen, Copenhagen, Denmark

**Keywords:** Non-inferiority, Acute malnutrition, Cluster randomized trial, Community-based management of acute malnutrition, Mid-upper arm circumference, Ready-to-use therapeutic food, Kenya, South Sudan

## Abstract

**Background:**

Acute malnutrition is a continuum condition, but severe and moderate forms are treated separately, with different protocols and therapeutic products, managed by separate United Nations agencies. The Combined Protocol for Acute Malnutrition Study (ComPAS) aims to simplify and unify the treatment of uncomplicated severe and moderate acute malnutrition (SAM and MAM) for children 6–59 months into one protocol in order to improve the global coverage, quality, continuity of care and cost-effectiveness of acute malnutrition treatment in resource-constrained settings.

**Methods/design:**

This study is a multi-site, cluster randomized non-inferiority trial with 12 clusters in Kenya and 12 clusters in South Sudan. Participants are 3600 children aged 6–59 months with uncomplicated acute malnutrition. This study will evaluate the impact of a simplified and combined protocol for the treatment of SAM and MAM compared to the standard protocol, which is the national treatment protocol in each country. We will assess recovery rate as a primary outcome and coverage, defaulting, death, length of stay, average weekly weight gain and average weekly mid-upper arm circumference (MUAC) gain as secondary outcomes. Recovery rate is defined across both treatment arms as MUAC ≥125 mm and no oedema for two consecutive visits. Per-protocol and intention-to-treat analyses will be conducted.

**Discussion:**

If the combined protocol is shown to be non-inferior to the standard protocol, updating guidelines to use the combined protocol would eliminate the need for separate products, resources and procedures for MAM treatment. This would likely be more cost-effective, increase availability of services, enable earlier case finding and treatment before deterioration of MAM into SAM, promote better continuity of care and improve community perceptions of the programme.

**Trial registration:**

ISRCTN, ISRCTN30393230. Registered on 16 March 2017.

**Electronic supplementary material:**

The online version of this article (10.1186/s13063-018-2643-2) contains supplementary material, which is available to authorized users.

## Background

Acute malnutrition is a major global public health problem affecting an estimated 52 million children under 5 years of age [1]. Of these, some 35 million have moderate acute malnutrition (MAM) and 17 million have severe acute malnutrition (SAM). The true burden of disease is however likely much higher. This is because most current estimates are based on cross-sectional survey data giving prevalence figures, whereas SAM and MAM, being short-lasting conditions, should ideally be assessed in terms of incidence [2, 3]. Malnutrition in all its forms is an underlying cause of 3.1 million (45%) of deaths among children under 5, with wasting alone responsible for 875,000 child deaths per year [4].

Despite the availability of effective, evidence-based treatment programmes, especially for SAM, their public impact is often limited by low coverage of the affected population. According to recent estimates, less than 20% of children with SAM receive the treatment they need [5]. Figures for MAM programme coverage are unknown—but are likely to be lower since it is often perceived to be less of a priority compared to SAM. This is despite wasting, the major manifestation of both SAM and MAM, being on a continuum, with the cut-off between the two defined statistically (based on weight-for-height/length standard deviations from the median) rather than by any distinguishable clinical changes.

In humanitarian settings (as well as many other non-emergency but fragile contexts where malnutrition is common), current international and national recommendations involve treating SAM and MAM in separate programmes, using separate protocols and separate products managed by two large but separate United Nations agencies. SAM is treated with ready-to-use therapeutic food (RUTF) in an outpatient therapeutic programme (OTP), with oversight and technical guidance from the United Nations International Children’s Fund (UNICEF). MAM is treated with ready-to-use supplementary food (RUSF) or fortified corn soy blend ++ (CSB++) in a supplementary feeding programme (SFP), with oversight and technical guidance from the World Food Programme (WFP). The shortcomings of this system include the following: (1) it is logistically complicated to implement, requiring the procurement of two different nutritional products and the set-up of two separate programs, in coordination with two separate UN agencies, (2) it is consequently expensive, and (3) it often results in the prioritization of SAM over MAM: many aid agencies and governments offer treatment only of SAM due to the challenges associated with procuring two products and coordinating two programmes. This results in a situation where treatment may not be available to children with MAM until they deteriorate to SAM.

**Fig. 1:**
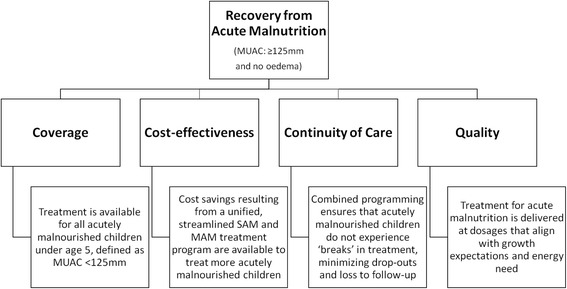
ComPAS conceptual framework

Responding to these current challenges, the Combined Protocol for Acute Malnutrition Study (ComPAS) will assess the effectiveness of a simplified, combined protocol for the treatment of uncomplicated SAM and MAM for children 6–59 months. The study aims to improve the quality, coverage, continuity and cost-effectiveness of care (Fig. 1). The combined protocol uses one product (RUTF) for both SAM and MAM, at doses designed to optimize growth and minimize cost at each stage of treatment. Admission and discharge is assessed using only mid-upper arm circumference (MUAC) and oedema [6]. Differences between the combined and standard protocol are presented in Table 1.

**Table 1:** Nutritional protocol for the control and intervention trial arms

	Standard protocol (control)	Combined protocol (intervention)
Admission criteria	OTP	• MUAC <125 mm and/or • Bilateral pitting oedema (+/++) and • Clinically uncomplicated^a^
	• WHZ <−3 and/or • MUAC <115 mm and/or • Bilateral pitting oedema (+/++) and • Clinically uncomplicated^a^	
	SFP	
	• Discharged from OTP and/or • WHZ <−2 to >−3 and/or • MUAC 115 to <125 mm and • Clinically uncomplicated^a^	
Treatment frequency	OTP	MUAC <115 mm and/or oedema (+/++)
	Weekly	
	SFP	MUAC 115 to <125 mm
	14 days	
Treatment transition criteria	▪ Child meets OTP ‘cured’ definition as described below	• Two consecutive MUAC measurements at or above 115 mm and • No oedema
Dosage	OTP	MUAC <115 mm and/or oedema (+/++)
	RUTF 200 kcal/kg/day	RUTF 1000 kcal/day (2 sachets/day)
	SFP	MUAC 115 to <125 mm
	RUSF 500 kcal/day (1 sachet/day)	RUTF 500 kcal/day (1 sachet/day)
Cured	OTP	≥125 mm for 2 consecutive measurements and no oedema
	▪ Child maintains MUAC ≥115 mm for 2 consecutive visits^b^ and/or ▪ WHZ >−3 for 2 consecutive visits^b^ and ▪ No oedema for 2 consecutive visits	
	SFP	
	Child maintains WHZ >−2 and/or MUAC ≥125 mm for a period of 2 consecutive visits^b^	

^a^Clinically uncomplicated: passes the appetite test, no Integrated Management of Childhood Illness (IMCI) danger signs [11]/no serious medical complications

^b^Dependent on which criteria the child was admitted on

ComPAS was inspired by increasing realization that combining the treatment of SAM and MAM makes biological sense—since wasting is a continuum condition rather than two distinct and different problems; operational and financial sense—since staff skill set and infrastructure are very similar for the two programmes and may thus usefully be shared; and public health sense—since a combined programme should be easier to access and should thus improve overall programme coverage.

Early data supporting the hypothesis of clinical non-inferiority comes from a 2013 Washington University trial in Sierra Leone [7]. This study explored the efficacy of an integrated SAM/MAM treatment protocol using one product (RUTF) but at different doses for children <115 mm (175 kcal/kg/day) and 115 to <125 mm (75 kcal/kg/day). Controls followed standard care guidelines: RUTF for SAM and SFP with CSB++ for MAM. Results showed that the integrated programme had a reduced caseload of SAM, due to earlier treatment of children presenting as MAM, with a similar recovery rate (83% vs. 79%) and higher coverage (71% vs. 55%, *p* = 0.0005). Children who received integrated management recovered more rapidly, with greater MUAC gain and higher weight-for-height *Z*-score (WHZ) upon discharge.

Other data supporting the use of a single nutritional product but at different doses comes from our own secondary analysis of routine nutrition programme data from five countries (Yemen, Pakistan, South Sudan, Chad, Kenya) and three different agencies (International Rescue Committee (IRC), Action Against Hunger—USA (ACF-USA), and Médecins Sans Frontières—France (MSF-France)) [8]. This analysis used observational data to assess the rate of growth of children recovering from acute malnutrition in OTPs and SFPs in order to determine energy requirements and propose an optimized dose of RUTF that correlates with the MUAC category. We found that:

- Growth trends in MUAC mirror those of proportional weight gain and rates of MUAC and weight gain slow with increasing MUAC.
- As the rates of MUAC and weight gain slow, proportional energy needs decrease.
- Total energy needs of 95% of all children with a MUAC <125 mm can be met with 1000 kcal/day.

Based on these observations, the combined and simplified MUAC-based dosage protocol was developed, and it is this which is being tested in the current study:

- Children with a MUAC <115 mm and/or oedema receive two sachets of RUTF per day (1000 kcal).
- Children with a MUAC 115 to <125 mm receive one sachet of RUTF per day (500 kcal).

The objective of our current project is to assess the effectiveness of a combined SAM/MAM protocol compared to standard care (separate SAM/MAM treatment) in two countries: Kenya and South Sudan. The outcomes of stage 2 are described in Table 2.

**Table 2:** Outcomes

	Measurement variable	Analysis metric	Method of aggregation	Time point
Primary				
Recovery	MUAC ≥125 mm and no oedema	Final value	Proportion	End of treatment
Secondary				
Coverage	% of children eligible for treatment (MUAC <125 mm) who receive it	Final value	Proportion	Mid-point of study
Defaulter	Child discharged as defaulter (3 missed visits)	Final value	Proportion	End of treatment
Died	Child died during treatment	Final value	Proportion	End of treatment
Length of stay	Days in treatment	Duration of time	Sum	End of treatment
Average daily weight gain	g/kg/day	Daily	Mean	End of treatment
Average daily MUAC gain	mm/day	Daily	Mean	End of treatment

We will add to the available evidence by (1) testing a dosing protocol based on the MUAC category, not weight [6, 9, 10]; (2) comparing against a control SFP using RUSF, instead of CSB++; (3) comparing equivalent definitions of recovery in both control and intervention groups; (4) using a larger sample size across two countries; and (5) conducting a thorough cost-effectiveness analysis using proven methodologies.

The publication of this study protocol aims to improve transparency and share information to support the development of similar studies that seek to simplify the protocols for acute malnutrition and increase the availability of treatment.

## Methods/design

### Trial design

The study is a multi-country cluster randomized controlled non-inferiority trial. The units of randomization are health facilities stratified by country and then randomly assigned to the control or intervention group. Children in the control group receive the standard protocol while those in the intervention group receive the combined protocol. The study includes a total of 24 clusters, 12 in each country.

### Hypothesis

We hypothesize that the combined protocol will be as effective as the standard protocol in the treatment of severe and moderate acute malnutrition as measured by recovery rate, length of stay, average weekly weight and MUAC gain, and coverage. The combined protocol will be more cost-effective than the standard protocol. Differences between the combined and standard protocols are presented in Table 2.

### Study site and population

There are two sites in this multi-center cluster randomized trial: Aweil East, South Sudan and Nairobi, Kenya. Aweil East is a rural setting in the former state of Northern Bahr el Ghazal with a total population of 309,921 and an under five population of 59,574 according to the most recent national population and housing census in 2008. ACF-USA supports 16 malnutrition clinics in the area; each is approximately 20–30 km apart. At the time the study was initiated, only 12 of these clinics were supported by ACF-USA, and these 12 were all selected for inclusion in the study.

Nairobi county is an urban area with a total population of approximately 3.1 million, of which 13% are children under 5 years, according to the 2009 population census. Three sub-counties of Nairobi were selected in collaboration with the Ministry of Health (MoH) based on a high burden of malnutrition and a need for nutritional support (Embakasi North, Embakasi South and Embakasi West). Out of the 32 health facilities in the three sub-counties, 12 health facilities were selected based on the following key factors: the level of care provided (hospitals and dispensaries excluded), the type of care provided (routine child health services available), the population served (slum or peri-urban communities), and expected caseload of malnutrition. Each treatment facility is approximately 3–5 km apart. The clinics in Nairobi are run by the MoH, and the IRC supports with research staff at each clinic to implement the ComPAS trial.

### Eligibility

Children 6–59 months with uncomplicated acute malnutrition are eligible for inclusion in the study per the following criteria:

- MUAC <125 mm

and/or

- Bilateral pitting oedema (+/++)

and

- Passes the appetite test (consumption of 30 g of RUTF within 20 min)

and

- No medical complications (i.e. no features of severe illness as defined by the Integrated Management of Childhood Illness (IMCI) [11], e.g. no severe nausea/vomiting, no severe dehydration, no severe pneumonia)

A child is excluded from the study if he or she has ever been enrolled in the ComPAS trial or if he or she is receiving SAM or MAM treatment elsewhere, unless he or she was recently discharged from SAM treatment in order to attend MAM treatment.

### Informed consent procedure

Consent is sought at two levels: at the level of the cluster, by the health facility officer in charge of each clinic prior to cluster randomization, and at the level of the individual, by the caretaker of each child prior to enrolment. When a caretaker arrives to any health facility included in the ComPAS trial seeking treatment for their malnourished child, they are seen by a community health or nutrition worker to confirm that they are eligible to receive treatment (i.e. they have a MUAC <125 mm, and/or a WHZ <−2, and/or oedema). Once their eligibility for treatment is confirmed, they are seen by the ComPAS research officer and clinical officer responsible for their care. First, the research officer will confirm that the present caretaker is a primary guardian for the child. If yes, the research officer describes the aims of the study as detailed in the ‘participant information sheet’, ensuring that the caretaker feels comfortable and understands the information. The participant information sheet provides details of the treatments provided in the study. The research officer will ensure the caretaker understands that treatment is available for all malnourished children regardless of whether they choose to participate in the study. Caretakers who agree to participate in the study will indicate their consent by signing a written consent form. If caretakers are not able to read or write, an impartial witness will oversee the consent process and attest to the caretaker’s verbal consent. If the caretaker is not a primary guardian or chooses not to participate in the study, their child is enrolled for treatment only, and their information is not collected.

### Randomization and blinding

#### Sequence generation

Randomization sequence is generated using an online sequence generator [12].

#### Type

There is stratification by country to ensure equal, 1:1 distribution of control and intervention clinics (clusters) in each country.

#### Allocation concealment mechanism and implementation

Clinics agree to participate understanding that group allocation is unknown in advance and is allocated randomly. The study statistician applies the random number sequence to a pre-written list of participating clinics, and the study team conveys the resultant allocation to the clinic staff. Individual carers attending clinics for the first time are very unlikely to know in advance whether their local clinic is in the intervention or control arm of the study.

#### Blinding

Due to the nature of intervention, this is not a fully blinded study. Front-line clinical and study staff must know whether they are treating children according to intervention or control (standard care) protocols, and the same staff enrol, manage and follow up patients. Staff in the standard protocol clinics were given refreshing trainings to reinforce standard protocol implementation, and staff in the combined protocol clinics were given trainings to roll out the new protocol. Staff in the clinics do not have access to overall treatment outcomes for each clinic or each arm of the trial; they are only aware of individual outcomes for the children they are responsible for treating. Similarly, individual carers cannot be blinded to the intervention being given to their child. Important to note however is that inter-group differences are unlikely to be striking to anyone but expert observers: both study arms use nutrient-dense food pastes, with RUTF and RUSF being peanut-based with similar taste and appearance; children are measured and clinically managed in exactly the same way in both study arms. The principal investigator is blinded to the treatment outcomes, in order to maintain objective trial management and analysis of results.

### Treatment

The combined protocol will be compared against the standard protocol (the national protocol in each country). The combined protocol admits all children with a MUAC <125 mm and/or oedema (+/++) and treats them according to a standardized dose of RUTF (children with a MUAC <115 mm or oedema receive 1000 kcal/day of RUTF; children with a MUAC 115 to <125 mm receive 500 kcal/day of RUTF). The combined protocol remains in line with globally accepted practice, with children recovering from SAM receiving enough therapeutic food to cover their total energy needs and children with MAM receiving a supplement to their family diet (most SFP protocols provide approximately 500–550 kcal/day of RUSF). The standard protocol includes treatment of SAM in an OTP using RUTF (200 kcal/kg/day) and MAM in a SFP using RUSF (500 kcal/day), as approved by the WHO, UNICEF, WFP and Ministries of Health in each country. The medical components of each protocol are the same, with children in the combined protocol with a MUAC <115 mm and/or oedema receiving the same systematic medications as those in the standard protocol enrolled in the OTP (per national guidelines). Children are managed at level 1 (community) health facilities by nurses or clinical officers for the medical aspects of care and by nutritionists for the nutritional components. If a child requires any additional medical care, they are seen by a nurse or clinical officer in the same facility. The few requiring higher level treatment are referred to the nearest inpatient facility. The qualifications of the staff and level of care are the same across both arms. The combined and standard nutritional treatment protocols are summarized in Table 1.

At each weekly follow-up visit, SAM cases are seen by a clinician (nurse or clinical officer) for the medical review and a nutritionist for the nutritional review. MAM cases differ in that they return for follow-up bi-weekly and receive a medical assessment only if they exhibit signs of illness or non-response to treatment. If a child has developed any medical complications or needs more specialist assessment, they are referred to the nearest inpatient facility (hospital in Nairobi, Kenya; Stabilization Centre in Aweil East, South Sudan). If a child is not gaining weight or mid-upper arm circumference appropriately, the nutritionist will discuss with a clinician based at the health facility, exploring possible underlying medical conditions, as well as counselling the caregiver to address possible contributory factors (e.g. sharing of RUTF/RUSF with other children, household food insecurity, breastfeeding for younger children). This is the same process across both arms.

Children who miss visits are followed up by a community health worker (Kenya) or community volunteer (South Sudan) to encourage caretakers to return. Children who default are followed up by a community health worker to ascertain the child’s true status (cured, died or remains malnourished).

Participants will be followed up 4 months post-discharge to assess nutritional status (weight, height, MUAC and oedema), health status (any hospitalizations since discharge and morbidities in the prior 2 weeks), and breastfeeding status. The objective of the follow-up study is to assess long-term impacts of the combined protocol.

**Fig. 2:**
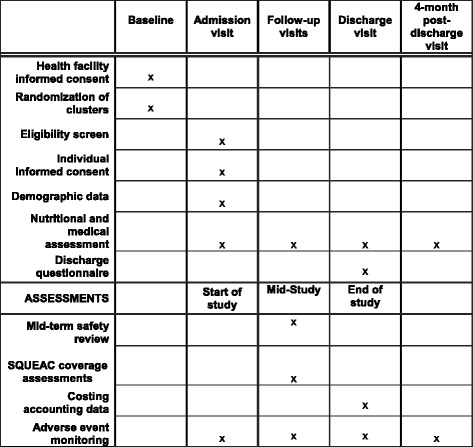
Schedule of enrolment, interventions and assessments

The timeline for enrolment, interventions and assessments is in Fig. 2.

### Outcomes

The primary outcome, ‘recovery’, in both the control and intervention groups is defined as two consecutive measurements with a MUAC ≥125 mm and no oedema. The secondary outcomes include coverage, rates of defaulting and death, length of stay, and average daily weight and MUAC gain. Cost-effectiveness will be assessed through an economic analysis of financial data following the study. The outcome metrics are described in Table 2.

### Quality control and supervision

A research officer is based in each of the 24 clinics included in this study. Each research officer oversees the consent process, enrolment and treatment of children according to the study protocol and data management. They participate in initial and refresher trainings with health facility staff as well as provide continuous on-the-job trainings to clinicians and community health volunteers involved in administering treatment according to the study protocol. They assess the accuracy of anthropometric measurements taken by health facility staff and review the patient cards and registers on a daily basis to ensure data quality.

Research officers are supervised by a team of roving senior supervisors, including senior research officers, deputy manager, research field coordinator and the nutrition coordinator.

### Data collection and management

In Kenya, patient anthropometry data is immediately transcribed from paper patient cards by the research officer based at the clinic using a digital data collection application (CommCare HQ; https://www.commcarehq.org) on Wi-Fi- and SIM card-enabled 7-in. Samsung Galaxy Tab A tablets. Due to Internet connectivity challenges in South Sudan, patient information is collected on paper first and later transferred to a 9.6-in. Samsung Galaxy Tab E tablet by a central data entry clerk. Data is reviewed weekly by the field coordinators and any discrepancies corrected and recorded using a digital ‘data error correction form’. In addition to the primary and secondary outcomes listed in Table 2, additional information is collected from the caregiver on morbidity, breastfeeding, protocol adherence, food security, hygiene and sanitation, caretaker education and demographic characteristics.

The procedures for assessing child anthropometry (weight, height, MUAC and oedema) are detailed in Additional file 1.

### Analysis

Sample size calculation was determined using an expected recovery rate of 85% based on the average programme statistics provided by the MoH in Nairobi, Kenya, and Action Against Hunger in Aweil East, South Sudan. If the combined protocol is non-inferior to the current protocol, allowing for a 10% non-inferiority margin, then we require 12 clusters in each arm with 100 children in each cluster to demonstrate non-inferiority of the combined protocol, with 80% power at the 5% level of significance. An intra-cluster correlation coefficient (ICC) of 0.05 was assumed, a conservative estimate based on the results of a similar cluster randomized study testing an integrated SAM/MAM protocol in Sierra Leone [7]. In order to account for losses to follow-up (estimated as 15%) and cross-overs (estimated as 5% in each arm), 150 children per cluster will be recruited for inclusion in the study. The cluster size calculation is as follows: 100 × 1/(1 − 0.15) × (1/(1 − 0.05 − 0.05)^2^) = 146. Therefore, with a cluster size of 150, and 24 clusters, 3600 children in total will be recruited in this study (1800 in each country).

**Fig. 3:**
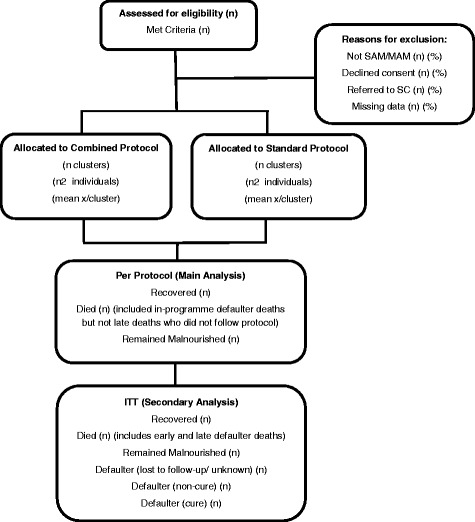
Analysis flow chart

Statistical analysis will be conducted at the individual level with appropriate adjustment for clustering within 24 clusters. Descriptive summaries of participant characteristics by arm will be tabulated. Descriptive statistics for continuous variables will include the mean, standard deviation, median, range and the number of observations. Categorical variables will be presented as numbers and percentages. The main analysis of the primary and secondary outcomes will be per-protocol given that this is a non-inferiority trial. Additionally, intention-to-treat analyses will be presented. Analyses are described in Fig. 3.

### Ethics

#### Confidentiality

If a caretaker consents for their child to be enrolled, they are issued a ComPAS ID number, which is affixed to their paper forms and entered in digital forms. All patient forms are maintained securely in a locked file throughout the study to ensure patient confidentiality. In Kenya, data collection tablets are password protected and research officers are assigned individual logins and passwords to access the digital data collection application (CommCare). Research officers are able to enter data for patients at their assigned facility only. After, the entered data is synced online to the CommCare platform, which occurs daily. Research officers cannot update prior entries unless they use an approved data error correction form. They are able to view a very limited selection of historical data for the purpose of patient identification and missed visit tracking. In South Sudan, data entry tablets are password protected and data entry clerks based in the ACF office have exclusive access to CommCare using individual logins.

#### Safety

Adverse events, including hospitalizations and deaths, are monitored and recorded by the research team for review by the independent trial safety committee (described in the ‘Trial governance’ section).

#### Ethical approval

This study protocol was approved by the following ethical review committees:

1. Kenya Medical Research Institute (KEMRI), Nairobi, Kenya. Reference: Non-KEMRI 551
2. Ministry of Health, Juba, South Sudan. Approved 21 November 2016
3. London School of Hygiene and Tropical Medicine, London, UK. Reference: 11826

### Trial governance

ComPAS is a research consortium of the International Rescue Committee, Action Against Hunger and the London School of Hygiene and Tropical Medicine. The trial is guided by a scientific committee of experts in paediatrics, humanitarian nutrition and epidemiology from the London School of Hygiene and Tropical Medicine, Washington University School of Medicine and the University of Copenhagen/University of Tampere. A trial safety committee, comprising an independent chair and a statistician from the London School of Hygiene and Tropical Medicine, will review the study outcomes and adverse events at the mid-point of the trial.

This trial is registered as ISRCTN30393230, 16 March 2017 (Additional file 2).

## Discussion

This study, a multi-site cluster randomized non-inferiority trial, expected to be completed by mid-2018, is evaluating the effectiveness of a simplified and combined treatment protocol for SAM and MAM against the standard protocol of separate products (RUTF, RUSF) and programmes (OTP, SFP) for SAM and MAM. If the results show that the combined protocol has non-inferior recovery rates compared to the standard protocol, this study will contribute to the evidence that current CMAM protocols can be simplified and that treatment of SAM and MAM can be combined (one product, one protocol and one anthropometric criterion for admission and discharge). This would make it easier for health care providers to offer treatment, reaching more children at an earlier stage before they deteriorate into severe malnutrition. The results of this trial should be interpreted together with the cost-effectiveness analysis to support policy decisions aimed at improving the coverage and quality of treatment.

## Trial status

Recruitment of trial participants began on 15 May 2017 and is expected to be completed by 1 June 2018, at which point data will be analysed. The SQUEAC coverage assessments will be complete in both countries by 25 February 2018.

## Additional files

Additional file 1: Procedures for taking anthropometric measurements. (DOCX 14 kb)

Additional file 2: SPIRIT checklist. (DOCX 52 kb)

## Abbreviations

ACF: Action Against Hunger
CMAM: Community-based management of acute malnutrition
ComPAS: Combined Protocol for Acute Malnutrition Study
CSB++: Corn soy blend ++ (fortified)
IRC: International Rescue Committee
ITT: Intention-to-treat
MAM: Moderate acute malnutrition
MSF: Médecins Sans Frontières
MUAC: Mid-upper arm circumference
OTP: Outpatient therapeutic programme
RUSF: Ready-to-use supplementary food
RUTF: Ready-to-use therapeutic food
SAM: Severe acute malnutrition
SC: Stabilization Centre
SFP: Supplementary feeding programme
SQUEAC: Semi-quantitative evaluation of access and coverage
UNICEF: United Nations International Children’s Fund
WFP: World Food Programme
WHO: World Health Organization
